# Identification of ion homeostasis-related genes as diagnostic biomarkers for pulmonary arterial hypertension via WGCNA and machine learning

**DOI:** 10.3389/fgene.2026.1823486

**Published:** 2026-09-24

**Authors:** Yang Chen, Qiu Chen, Senzhong Zheng

**Affiliations:** Thoracic Surgery, Taizhou First People’s Hospital, Taizhou, Zhejiang, China

**Keywords:** diagnostic biomarker, ion homeostasis, machine learning, pulmonary arterial hypertension, WGCNA

## Abstract

**Background:**

Pulmonary arterial hypertension (PAH) is a severe cardiovascular disease, with early diagnosis being difficult. Ion homeostasis imbalance contributes to PAH pathogenesis, but the systemic regulatory network involving ion homeostasis-related genes (IHRGs) and their diagnostic potential remain unclear.

**Methods:**

This study utilized WGCNA and machine learning to identify potential PAH diagnostic biomarkers. WGCNA was applied to GSE117261 to identify IHRGs associated with PAH, followed by functional analysis via GO and KEGG pathways. Three machine learning algorithms—LASSO, SVM-RFE, and Boruta—were used to screen core genes, validated in GSE117261 and GSE113439 datasets. Additionally, bioinformatics analysis explored the genes’ roles in PAH’s immune microenvironment and regulatory mechanisms, complemented by *in vitro* hypoxia experiments validating the protective effects of S1PR1 and CA2 on endothelial cells.

**Results:**

WGCNA identified 55 IHRGs enriched in ion homeostasis and immune pathways. Machine learning cross-validation narrowed down 9 core genes, with ABCG2, CA2, and S1PR1 showing strong diagnostic performance. *In vitro* experiments further confirmed that CA2 expression was significantly upregulated under hypoxia, whereas S1PR1 was downregulated. S1PR1 overexpression effectively suppressed endothelial cell apoptosis, mitigated inflammatory responses, and enhanced tight junction protein expression, while CA2 knockdown produced opposing effects.

**Conclusion:**

This study identified ABCG2, CA2, and S1PR1 as potential diagnostic biomarkers for PAH, suggesting that IHRGs contribute to disease progression through modulating ion homeostasis and endothelial function. Among these, S1PR1 exhibited a definitive endothelial protective role, whereas CA2 may participate in hypoxia-induced endothelial adaptation, providing novel candidate targets for mechanistic research and early diagnosis of PAH.

## 1 Introduction

Pulmonary arterial hypertension (PAH) is a malignant cardiovascular disease characterized by a progressive increase in pulmonary vascular resistance and right ventricular afterload, ultimately leading to right heart failure (RHF) and death (Ruopp and Cockrill, 2022; Humbert et al., 2023). Its pathophysiological mechanisms are exceedingly complex, encompassing multiple facets such as pulmonary vascular endothelial cell dysfunction, abnormal proliferation and migration of smooth muscle cells (SMC), inflammatory immune responses, metabolic reprogramming, and extracellular matrix remodeling (Shen et al., 2024; Evans et al., 2021; Rajagopal and Yu, 2022). Although the clinical application of targeted drugs, including endothelin receptor antagonists, prostacyclin analogs, and phosphodiesterase-5 inhibitors, has improved the condition of some patients in recent years (Shah et al., 2023; Tang et al., 2022), the early diagnosis of PAH remains a significant challenge. Currently, the gold standard for PAH diagnosis still relies on right heart catheterization, an invasive procedure that is difficult to implement widely for early screening and dynamic monitoring (Olsson et al., 2023). Conversely, non-invasive indicators such as pulmonary artery pressure estimated by echocardiography and brain natriuretic peptide levels have insufficient sensitivity and specificity, particularly in the early stages of the disease (Arshad and Duarte, 2021; Topyla-Putowska et al., 2021). Therefore, exploring novel molecular diagnostic biomarkers with high sensitivity and specificity is of critical clinical significance for achieving early warning, precise classification, efficacy evaluation, and prognostic assessment of PAH.

In the pathogenesis and progression of PAH, the dynamic balance of intracellular and extracellular ion concentrations, also called ion homeostasis, plays a fundamental role (Balistrieri et al., 2023; Coste and Delmas, 2024). Ions such as potassium, calcium, sodium, and chloride are precisely regulated at the plasma membrane through corresponding channels, transporters, and pumps, directly influencing the membrane potential, excitation-contraction coupling, proliferation, and apoptosis of pulmonary artery SMC (Redel-Traub et al., 2022; Chen et al., 2021; Liu and Lin, 2022). Research by Hiraishi et al. (2022) demonstrated that TRPM7 plays a key role in PAH progression by regulating vascular cell phenotypic transformation and proliferation, holding clinical diagnostic value. Furthermore, the calcium-activated chloride channel ANO1 (TMEM16A) is central to the regulation of vascular smooth muscle tone in various vessels (Jimenez et al., 2022), while aberrant activation of calcium signaling pathways serves as a crucial hub driving SMC proliferation and vascular remodeling (Balistrieri et al., 2023). Ion homeostasis within organelles such as mitochondria and the endoplasmic reticulum is also closely associated with processes including cellular metabolism, oxidative stress, and apoptosis (Zheng et al., 2023; Delisi and Saatloo, 2023). However, current research on ion homeostasis-related genes (IHRGs) in PAH has largely focused on individual or a few molecules, lacking a systematic biology perspective to comprehensively elucidate the synergistic patterns of IHRGs within the PAH pathological network. Meanwhile, which IHRGs act as core drivers in the disease progression and whether they possess the potential to be translated into biomarkers for clinical diagnosis, remain urgent scientific questions to be addressed.

This study employed Weighted Gene Co-expression Network Analysis (WGCNA) to systematically identify IHRG modules significantly associated with the clinical features of PAH. By further integrating multiple machine learning algorithms, such as LASSO regression, Boruta, and Support Vector Machine-Recursive Feature Elimination (SVM-RFE), core genes with diagnostic value were screened. The efficacy of these core genes in the early identification and differential diagnosis of PAH was evaluated through validation in independent cohorts and experimental studies. Ultimately, this research aimed to reveal the translational potential of IHRGs as novel non-invasive diagnostic biomarkers, offering new insights for the precise diagnosis and treatment of PAH.

## 2 Materials and methods

### 2.1 Data download

Transcriptomic data related to PAH were obtained from the Gene Expression Omnibus (2026) database. Among the datasets, GSE117261, comprising 58 PAH samples and 25 normal control samples, was designated as the training set and used for subsequent WGCNA. The dataset GSE113439, containing 15 PAH samples and 25 normal control samples, served as an independent validation set for subsequent analyses.

IHRGs were obtained from the Harmonizome database (Harmonizome, 2024).

### 2.2 Identification and characterization of PAH-IHRGs

A gene co-expression network was constructed for the GSE117261 dataset using the *WGCNA* package (Langfelder and Horvath, 2008). Genes ranked in the top 20% by variance in expression across the dataset were selected as input data, with sample grouping information serving as the phenotypic trait. The optimal soft-thresholding power was determined to be 6 using the pickSoftThreshold function, and the minimum module size was set to 50. Subsequently, the blockwiseModules function was employed to construct a gene cluster dendrogram. Branches of the dendrogram were merged into distinct gene modules using the dynamic tree-cutting algorithm. Subsequently, a heatmap analysis was generated to visualize the correlations between each gene module and the PAH phenotype, thereby identifying key gene modules significantly associated with disease progression.

Genes within the PAH-related modules were intersected with the set of IHRGs to obtain the PAH-IHRGs. Subsequently, Gene Ontology (GO) and Kyoto Encyclopedia of Genes and Genomes (KEGG) enrichment analyses were performed on the PAH-IHRGs using the *clusterProfiler* package (Wu et al., 2021). The enrichment results were visualized using the ggplot2 package (2016).

### 2.3 Identification of diagnostic genes using machine learning algorithms

First, the SVM-RFE algorithm from the caret package (2008) was used to screen for potential diagnostic genes by progressively eliminating redundant features. Concurrently, feature importance was evaluated using the *Boruta* package (Kursa and Rudnicki, 2010) to identify candidate biomarkers significantly associated with the PAH phenotype. Furthermore, a LASSO regression model was constructed using the *glmnet* package (Friedman et al., 2010), and the optimal penalty parameter (λ.min = 0.01772027) was determined through 10-fold cross-validation (CV), thereby extracting key genes with non-zero coefficients. Finally, by integrating the screening results of the three algorithms and performing an intersection analysis, the core diagnostic genes for subsequent validation were identified.

### 2.4 Immune correlation analysis of diagnostic genes

First, the single-sample gene set enrichment analysis (ssGSEA) method was used to quantify the enrichment levels of immune cell subsets and immune-related functions in samples from healthy controls and PAH patients. Subsequently, Spearman correlation analysis was performed to evaluate the associations between the expression of core diagnostic genes and the infiltration levels of various immune cells. The correlation results were visualized as a heatmap using the *ggplot2* package to intuitively illustrate the association patterns between diagnostic genes and the immune microenvironment.

### 2.5 Gene set enrichment analysis (GSEA)

Based on the expression levels of the diagnostic genes, patient samples were divided into high-expression and low-expression groups using the median gene expression value as the cutoff. Subsequently, GSEA was performed on PAH samples from each group using *GSEA* software (version 4.3.3), with the KEGG database (KEGG, 2025) as the reference.

### 2.6 Prediction of microRNAs (miRNAs) and transcription factors (TFs) for diagnostic genes

Using the NetworkAnalyst online platform (NetworkAnalyst, 2019), we constructed a competing endogenous RNA (ceRNA) regulatory network diagram for the diagnostic genes based on transcriptional regulation and gene interaction information provided by the RegNetwork repository (RegNetwork, 2015). This was done to systematically reveal their potential post-transcriptional regulatory mechanisms.

### 2.7 GeneMANIA

Based on the GeneMANIA database (GeneMANIA, 2010), we constructed a protein-protein interaction (PPI) network of the diagnostic genes to identify their functionally related genes and interaction relationships. Furthermore, functional enrichment analysis was performed on this gene set to systematically explore its biological significance.

### 2.8 Cell culture

Human Pulmonary Great Artery Endothelial Cells (HPAEC) were purchased from Procell System (CP-H002, Procell system, CHINA). The cells were cultured in a dedicated complete HPAEC medium (CM-H002, Procell system, CHINA) under normoxic conditions at 37 °C with 5% CO_2_. A hypoxic cell model was established by culturing the cells under conditions of 1% O_2_, 5% CO_2_, and 94% N_2_.

### 2.9 Cell transfection

Cell transfection was performed using Lipofectamine 3000 (13778100, Thermo Fisher, USA). The S1PR1 overexpression plasmid (oe-S1PR1) and CA2 interference plasmid (si-CA2) were designed and synthesized by GenePharma (Shanghai, China). Specifically, the full-length coding sequence (CDS) of human S1PR1 was cloned into the eukaryotic expression vector pcDNA3.1 (+) to construct the S1PR1 overexpression plasmid, with the empty vector pcDNA3.1 (+) serving as the negative control (oe-NC). Targeted specific siRNA was designed against the mRNA sequence of human CA2, and the sense strand sequence was 5′-UUA​CGG​AAU​UUC​AAC​ACC​UGC-3’; non-targeting siRNA was used as the negative control (si-NC).

For transfection, plasmid DNA or siRNA was incubated with Lipofectamine 3000 reagent in Opti-MEM medium (31985070, Gibco, USA) to form transfection complexes, which were subsequently added to the HPAEC cell culture system. At 48 h post-transfection, quantitative real-time PCR (qRT-PCR) was performed to detect the mRNA expression levels of S1PR1 and CA2 for verification of overexpression or knockdown efficiency, and the treated cells were subjected to subsequent experimental analyses.

### 2.10 qRT-PCR

Total RNA was extracted from cells in each treatment group using Trizol (15596018CN, Thermo Fisher, USA), and its concentration and purity were measured with a NanoDrop spectrophotometer. Subsequently, RNA was reverse transcribed into cDNA using a cDNA reverse transcription kit (D7168, Beyotime, China). qRT-PCR analysis was performed on an Applied Biosystems 7500 PCR system using the SYBR Green real-time PCR kit (D7265, Beyotime, China). The primer sequences are shown in Table 1.

**TABLE 1:** qRT-PCR primers.

Gene	Forward primer (5′-3′)	Reverse primer (5′-3′)
S1PR1	GACCCCGACTCGAGCTGC	GAC​GTA​GTC​AGA​GAC​CGA​GC
TNF-α	CTC​GAA​CCC​CGA​GTG​ACA​AG	TCA​GCT​TGA​GGG​TTT​GCT​ACA
IL-1β	CCA​AAC​CTC​TTC​GAG​GCA​CA	ATG​GCT​GCT​TCA​GAC​ACT​TGA
IL-6	CCA​ATC​TGG​ATT​CAA​TGA​GGA​GAC	AAT​GAG​GAC​ACA​CCC​ACC​TTT
CA2	CCC​CTG​TTG​ACA​TCG​ACA​CT	TCC​CTT​GAG​CAC​TGC​TTT​GT
β-Actin	ACA​GAG​CCT​CGC​CTT​TGC​C	GAT​ATC​ATC​ATC​CAT​GGT​GAG​CTG​G

### 2.11 Western blot (WB)

Cells in each group were lysed using RIPA buffer (P0013B, Beyotime, China) to extract total protein. Protein concentrations were determined using a BCA kit (P0009, Beyotime, China). Proteins from each sample were separated by gel electrophoresis and transferred onto PVDF membranes. The membranes were blocked with 5% skim milk at room temperature for 2 h, followed by overnight incubation at 4 °C with primary antibodies against Cleaved-Caspase 3 (1:1,000, Rabbit, 9661T, CST, USA), Bcl-2 (1:1,000, Rabbit, 26593-1-AP, Proteintech, USA), ZO-1 (1:1,000, Rabbit, 21773-1-AP, Proteintech, USA), Claudin-5 (1:1,000, Rabbit, 29767-1-AP, Proteintech, USA), and β-actin (1:8,000, Rabbit, 20536-1-AP, Proteintech, USA). After three washes with TBST, the membranes were incubated with Goat Anti-Rabbit HRP (1:8,000, 111–035–003, Jacson, USA) for 2 h at room temperature. Following removal of unbound secondary antibodies with TBST washes, protein bands were visualized using an ECL kit (34,577, Thermo Fisher, USA). The expression levels of target proteins were captured and analyzed using an imaging system.

### 2.12 Flow cytometry

Apoptosis of HPAEC cells was detected using an Annexin V-FITC/PI Apoptosis Detection Kit (560,931, BD Biosciences, USA). First, single-cell suspensions were prepared from each sample. Subsequently, 500 μL of the cell suspension was mixed with 5 μL of Annexin V-FITC and 5 HL of PI, and incubated for 15 min in the dark. Finally, the apoptosis rate of these cells was measured using a FACScan flow cytometer (BD Biosciences, USA).

### 2.13 Data analysis and statistical methods

For statistical analysis of *in vitro* experiments, experimental data were presented as mean ± standard deviation (SD). Comparisons between two groups were performed using Student’s t*-test*, provided the data met the assumptions of normal distribution and homogeneity of variance. Comparisons among more than two groups were conducted using one-way analysis of variance (ANOVA), followed by Tukey’s *post hoc* test for multiple comparisons between groups. All statistical analyses were performed using *R* software (version 4.2.1) and *GraphPad Prism* software (version 10.1.2).

## 3 Result

### 3.1 Identification and functional analysis of PAH-ihrg-related modules based on WGCNA

**FIGURE 1:**
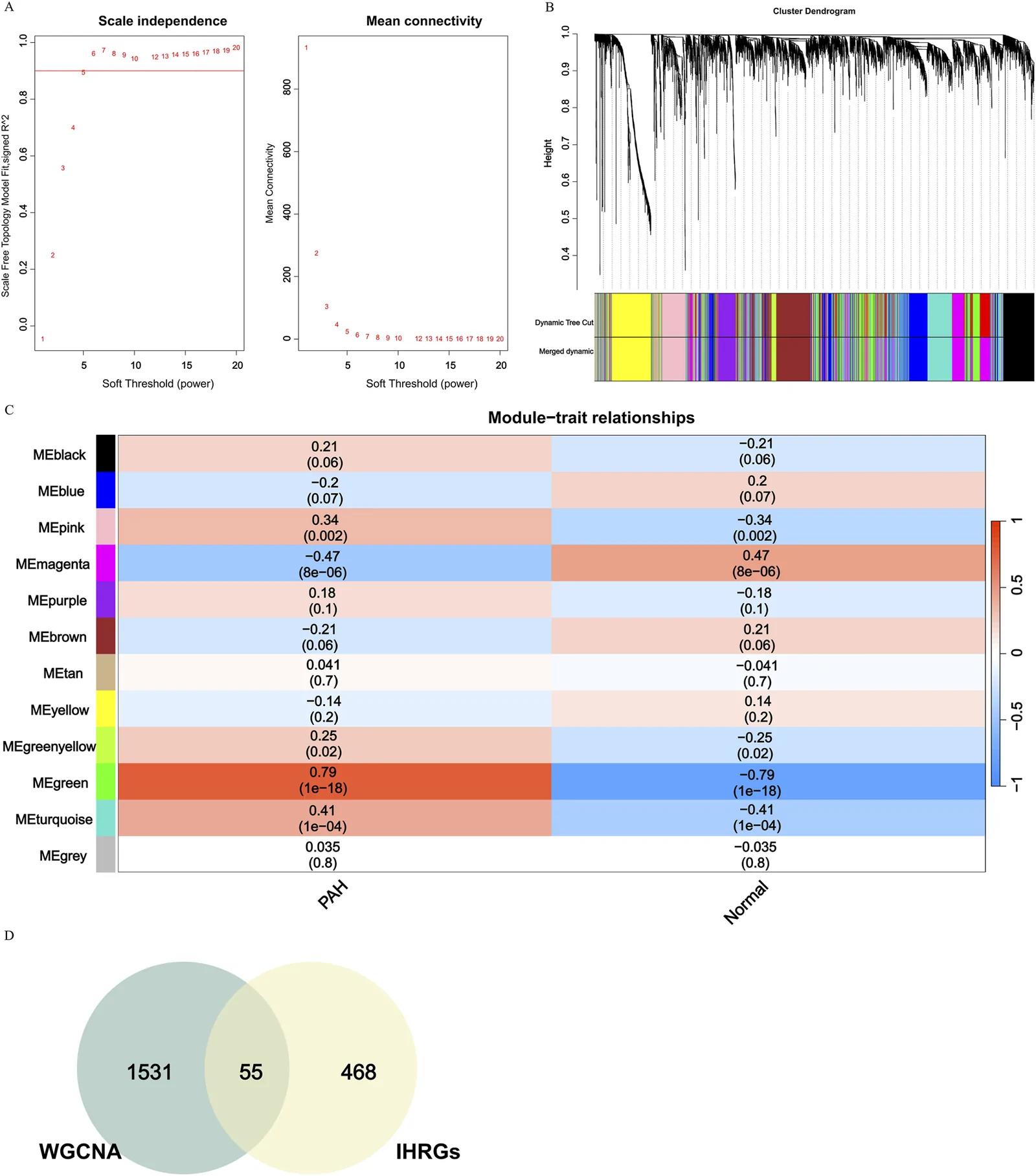
Identification of PAH-associated IHRGs modules and key module screening using WGCNA. **(A)** Scale-free topology fit indices and soft-thresholding power selection results for the gene co-expression network, demonstrating adherence to a scale-free distribution (R^2^ > 0.9). **(B)** The result of hierarchical clustering for the gene module division, identifying 12 co-expression modules, with distinct colors representing different modules. **(C)** Correlation heatmap depicting relationships between module eigengenes (ME) and PAH phenotypes, showing MEgreen, MEturquoise, and MEpink as significantly associated with PAH, with MEgreen exhibiting the strongest correlation. **(D)** Venn diagram illustrating the overlap of genes from the MEgreen, MEturquoise, and MEpink modules with the IHRGs gene set for the selection of PAH-related candidate genes.

**FIGURE 2:**
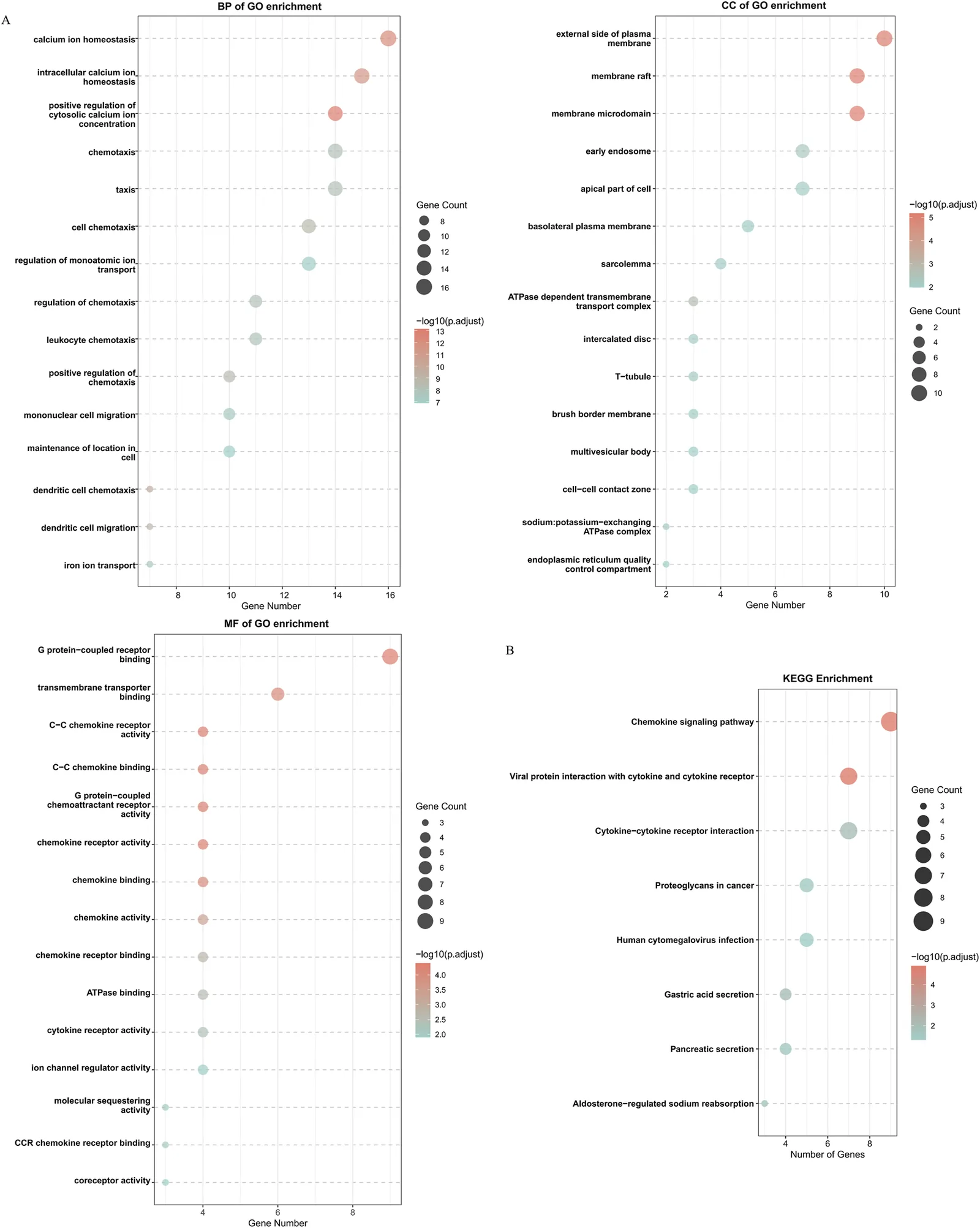
Functional analysis of PAH-IHRGs. **(A)** GO functional enrichment analysis of candidate genes. Bubble size indicates the number of genes enriched in each pathway, and color represents the *p*-value. **(B)** KEGG functional enrichment analysis of candidate genes.

To systematically construct the gene regulatory network of PAH, we analyzed the GSE117261 dataset using the WGCNA algorithm. Under the condition satisfying the scale-free topology criterion (R^2^ > 0.9, β = 6), a total of 12 gene co-expression modules were identified (Figures 1A,B). Correlation analysis revealed that the MEturquoise, MEgreen, and MEpink modules exhibited significant positive correlations with PAH. Among these, the MEgreen module showed the strongest correlation (R = 0.79, *P* < 0.0001), while the MEturquoise module (R = 0.41, *P* < 0.0001) and MEpink module (R = 0.34, *P* < 0.0001) also demonstrated significant correlations (Figure 1C). Subsequently, these three modules were merged and subjected to an intersection analysis with the IHRG set, yielding a total of 55 candidate genes (Figure 1D) (Supplementary Table S2). To further explore the functions of the candidate genes, enrichment analysis was performed. The results of GO enrichment analysis indicated that the candidate genes were significantly enriched in biological processes related to the regulation of calcium ion homeostasis, chemotaxis, and monoatomic ion transmembrane transport. Their molecular functions were primarily involved in chemokine and chemokine receptor binding and activity, G protein-coupled receptor binding, and ion channel regulation. Regarding cellular components, the enrichment was prominent in the cell membrane and its functional subdomains (such as membrane rafts, T-tubules, and brush border membranes), as well as various transmembrane transporter complexes, revealing the systematic role of the target gene set in coordinating ion homeostasis and immune cell directional migration (Figure 2A). Furthermore, KEGG enrichment analysis showed that the candidate genes were significantly enriched in immune and inflammation-related pathways, such as chemokine signaling transduction and cytokine-cytokine receptor interaction (Figure 2B), suggesting that these genes may be involved in immune regulation and the maintenance of internal environment homeostasis.

### 3.2 Identification of PAH-IHRGs as diagnostic biomarkers based on machine learning

**FIGURE 3:**
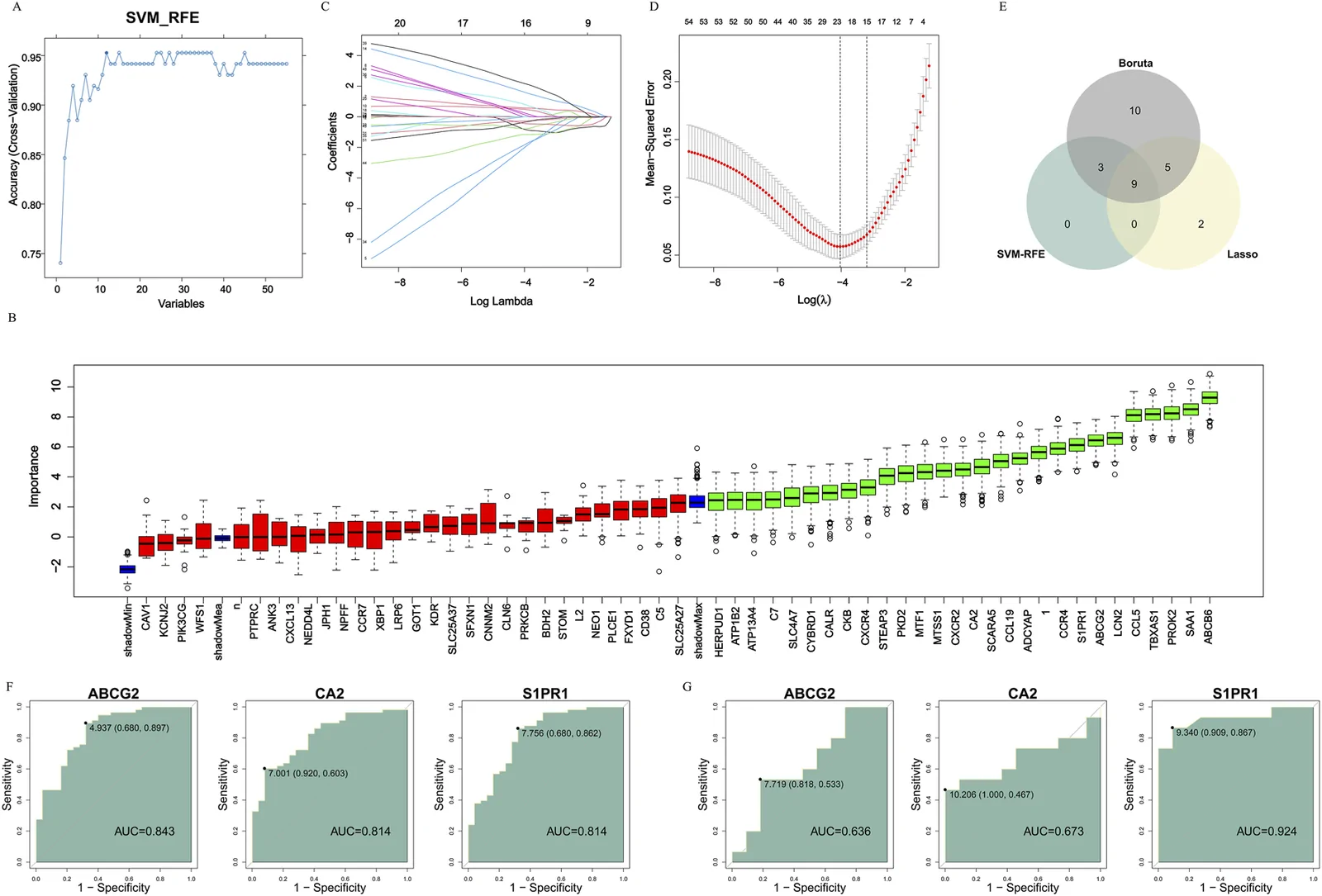
Identification of PAH-IHRGs diagnostic biomarkers based on machine learning. **(A)** 10-fold CV error curve showing the relationship between the number of genes fitted by the SVM-RFE algorithm and the SVM-RFE model error. **(B)** Feature variable selection results based on the Boruta algorithm. **(C)** LASSO coefficient spectrum showing the trend of gene coefficients under different λ values. **(D)** Cross-validation for tuning parameter selection in LASSO regression analysis. **(E)** Venn diagram illustrating the intersection of feature genes selected by the SVM-RFE algorithm and the Boruta algorithm. **(F)** ROC curves of ABCG2, CA2, and S1PR1 in the GSE117261 dataset. **(G)** ROC curves of ABCG2, CA2, and S1PR1 in the GSE113439 dataset.

To screen for genes with diagnostic value, this study employed three machine learning methods to systematically analyze the 55 candidate genes. First, based on the SVM-RFE method, 12 key genes were selected by progressively eliminating redundant features (Supplementary Table S3) (Figure 3A). Subsequently, the Boruta algorithm was used to assess the importance of all feature genes, identifying 27 candidate genes significantly associated with PAH (Supplementary Table S4) (Figure 3B). Finally, LASSO regression was applied to further reduce feature dimensions, with the optimal penalty parameter (λ = 0.01772027) determined through 10-fold CV, ultimately retaining 16 feature genes with non-zero coefficients (Supplementary Table S5) (Figures 3C,D). By integrating the screening results of the three algorithms and taking the intersection, a total of 9 common core diagnostic genes were obtained (Supplementary Table S6) (Figure 3E). To further refine the selection of key genes with stable and high diagnostic value, ROC curve analyses were performed for the nine candidate genes across two independent validation datasets (GSE117261 and GSE113439). The evaluation incorporated comparisons of AUC values and cross-cohort consistency. Only ABCG2, CA2, and S1PR1 exhibited relatively stable and robust diagnostic performance in both datasets. In the GSE117261 dataset, ABCG2, CA2, and S1PR1 exhibited high diagnostic value, with area under the curve (AUC) values of 0.843, 0.814, and 0.814, respectively (Figure 3F). In the GSE113439 dataset, these three genes also maintained stable diagnostic performance, with corresponding AUC values of 0.636, 0.673, and 0.924, respectively (Figure 3G). Collectively, ABCG2, CA2, and S1PR1 demonstrated stable diagnostic performance across different independent validation datasets, suggesting their promising potential for reliable differentiation of PAH.

### 3.3 Analysis of regulatory networks and functional characteristics of diagnostic genes

**FIGURE 4:**
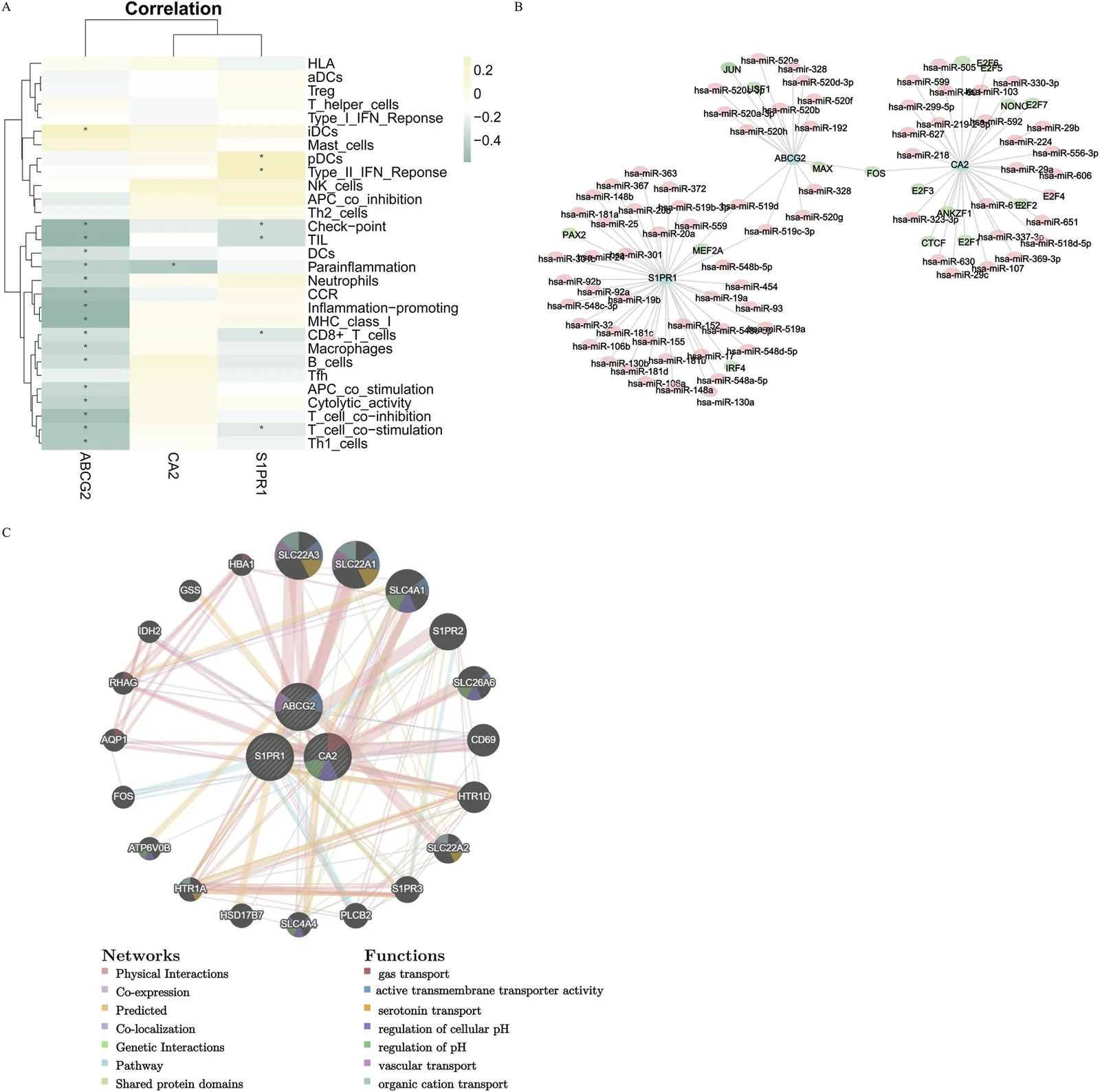
Analysis of regulatory networks and functional characteristics of diagnostic genes. **(A)** Heatmap showing the correlation analysis of ABCG2, CA2, and S1PR1 with immune cells. **(B)** Regulatory network analysis of ABCG2, CA2, and S1PR1. **(C)** Functionally similar genes and functional enrichment analysis of ABCG2, CA2, and S1PR1. *, *P* < 0.05.

To further explore the role of diagnostic genes in immune regulation and their underlying mechanisms, we first analyzed the correlation between these genes and immune characteristics. The expression level of S1PR1 was significantly positively correlated with the number of pDC cells and type II interferon response (*P* < 0.05), while it was significantly negatively correlated with immune checkpoint pathways, tumor-infiltrating lymphocytes, CD8^+^ T cells, and T cell co-stimulation (*P* < 0.05). The expression of ABCG2 was negatively correlated with the CCR signaling pathway, inflammation-promoting responses, infiltration of various immune cells (including macrophages and B cells), and immune checkpoint molecules (*P* < 0.05). The expression of CA2 showed a significant negative correlation with para-inflammatory responses (*P* < 0.05) (Figure 4A). Subsequently, we systematically predicted the upstream TFs and miRNA regulatory networks of the related genes. The transcription of S1PR1 might be regulated by PAX2, MEF2A, and IRF4, and it is potentially associated with multiple miRNAs. The expression of ABCG2 may be co-regulated by TFs such as MAX, JUN, and USF1, as well as miRNAs like hsa-miR-328 and hsa-miR-129. Meanwhile, CA2 might form a complex regulatory network involving miRNAs such as hsa-miR-618, hsa-miR-337–3P, and hsa-miR-224, along with TFs including NONO, E2F3, E2F2, FOS, and ANKZF1 (Figure 4B). To systematically elucidate their biological functions, this study constructed a PPI network using the GeneMANIA database and identified 20 core genes closely functionally associated with the diagnostic genes. Enrichment analysis of this network indicated that the related genes were primarily involved in maintaining cellular material exchange and internal environment homeostasis, encompassing key biological processes such as transmembrane transport, metabolite transport, and physiological homeostasis regulation (Figure 4C).

### 3.4 GSVA enrichment analysis of S1PR1

**FIGURE 5:**
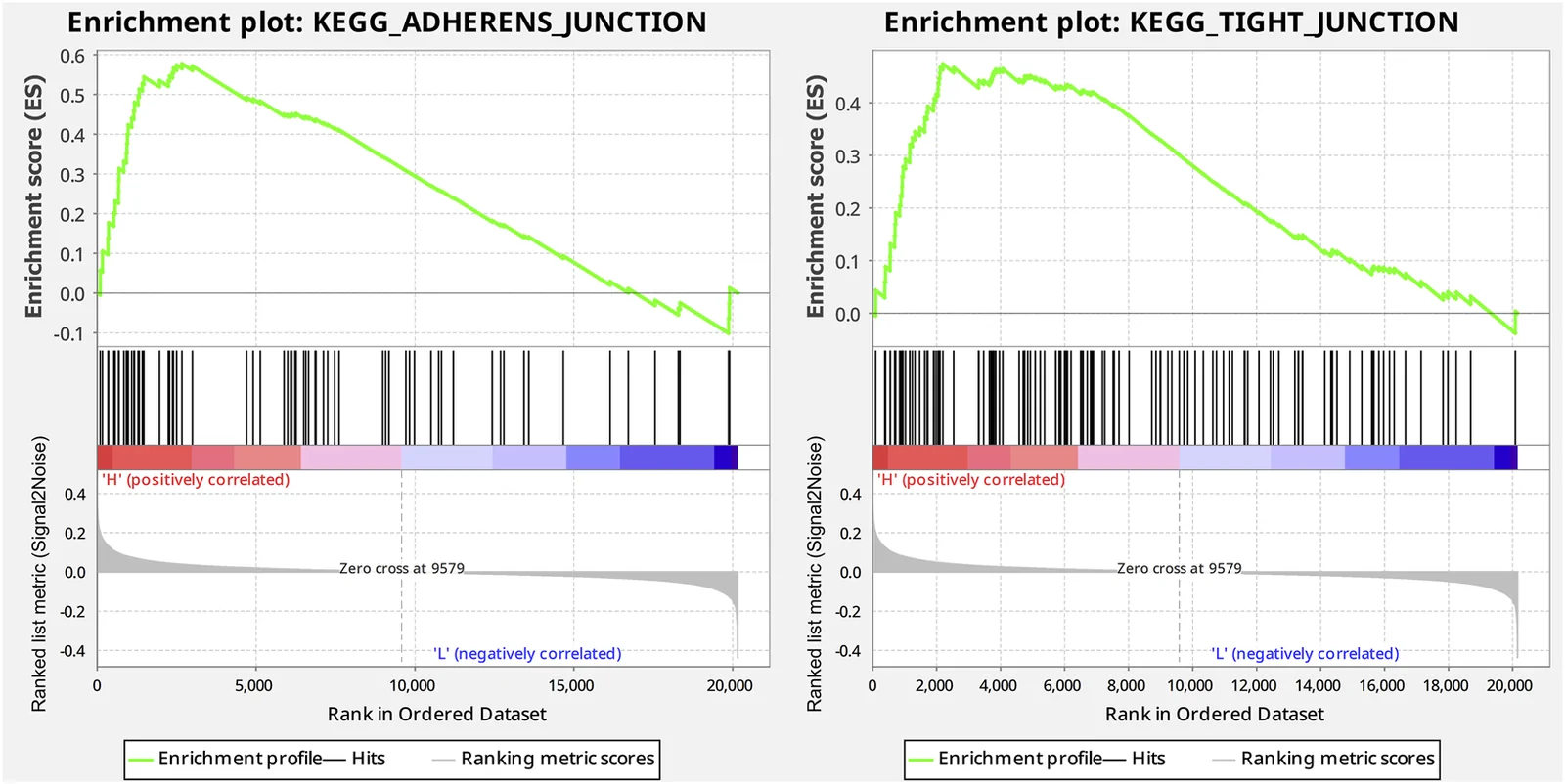
GSVA enrichment analysis of S1PR1.

To further elucidate the potential role of S1PR1 in the pathological process of PAH, we performed a functional enrichment analysis of its related signaling pathways using GSVA. The expression changes of S1PR1 were significantly enriched in both tight junction and adherent junction pathways (Figure 5). This finding suggested that S1PR1 may participate in the regulation of pulmonary artery endothelial barrier function and vascular remodeling by affecting cell junction-related pathways, thereby playing a role in the development of PAH. Previous studies have also linked the S1PR1 signaling axis in PAH to endothelial barrier dysfunction and to autoimmune-related mechanisms and vascular remodeling networks (Gluschke et al., 2022; Tran et al., 2021).

### 3.5 Effects of S1PR1 and CA2 on endothelial cell biologic functions under hypoxic conditions

To experimentally elucidate the biological roles of key genes in PAH-associated endothelial injury, subsequent *in vitro* investigations prioritized S1PR1 and CA2. Notably, while machine learning and GSVA identified S1PR1 as a stable core gene implicated in endothelial barrier integrity, systematic evidence regarding its functional response to hypoxia remains scarce, necessitating rigorous experimental verification. Concurrently, CA2, a molecule with marked differential expression in our screening and known involvement in ion homeostasis and pH regulation, has not been fully characterized regarding its role in PAH-related endothelial function. Although ABCG2 also fell within the core candidate gene set, its functions in transmembrane transport and drug efflux are well-established, with existing mechanistic studies in PAH (Shi et al., 2011; Gaskill et al., 2016). Given that functional validation of ABCG2 entails more complex transport systems and pharmacological models, this gene was not prioritized for *in vitro* functional experiments in the current study.

**FIGURE 6:**
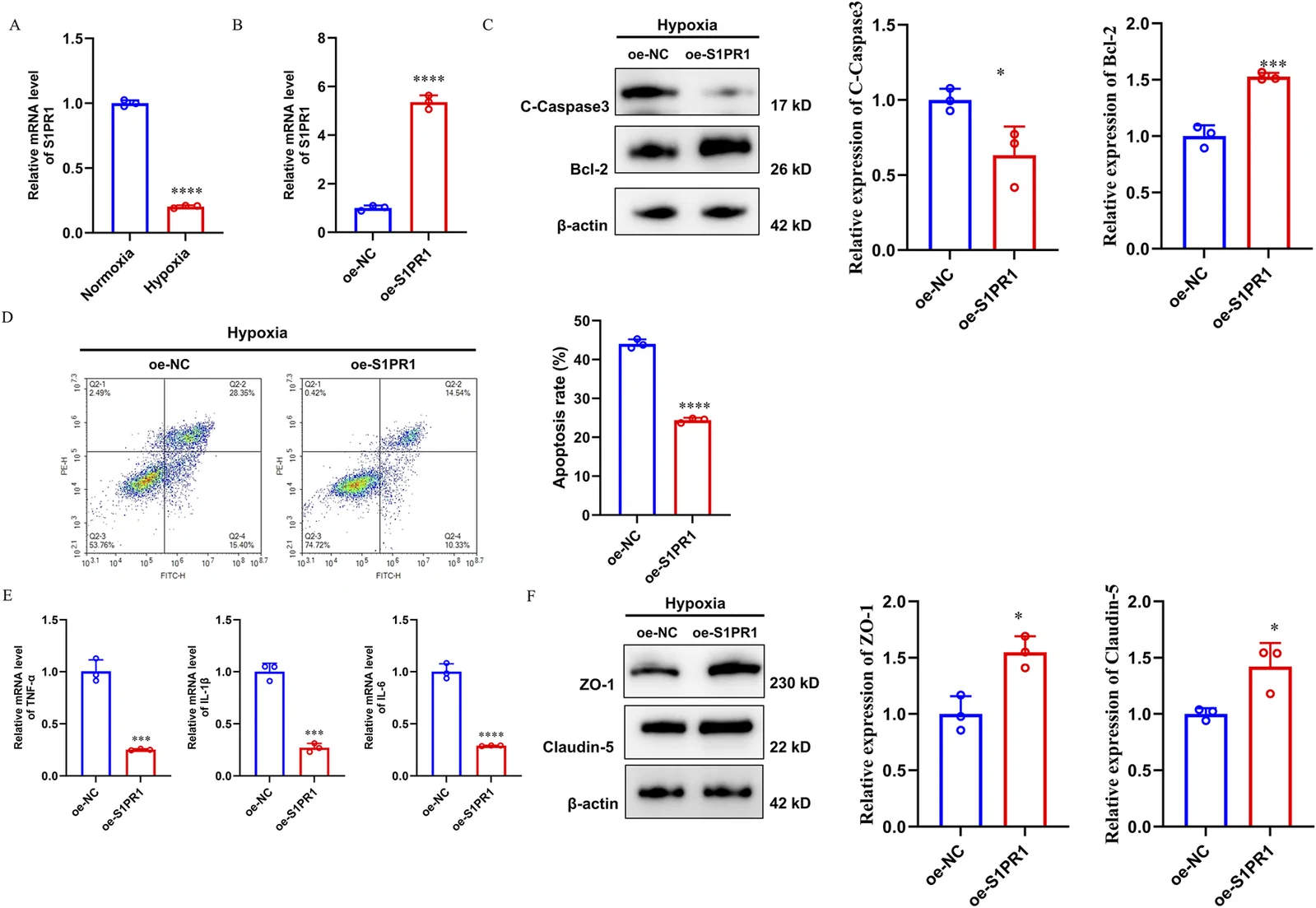
Effects of S1PR1 on endothelial cell biologic functions under hypoxic conditions. **(A)** qRT-PCR detection of S1PR1 expression levels in HPAECs under hypoxic conditions. **(B)** qRT-PCR detection of S1PR1 overexpression efficiency. **(C)** WB analysis of the effects of S1PR1 overexpression on Cleaved-Caspase 3 and Bcl-2 expression levels under hypoxic conditions. **(D)** Flow cytometry analysis of the effects of S1PR1 overexpression on apoptosis under hypoxic conditions. **(E)** qRT-PCR detection of the effects of S1PR1 overexpression on TNF-α, IL-1β, and IL-6 expression levels under hypoxic conditions. **(F)** WB analysis of the effects of S1PR1 overexpression on ZO-1 and Claudin-5 expression levels under hypoxic conditions. *, *p* < 0.05; ***, *p* < 0.001; ****, *p* < 0.0001.

An *in vitro* hypoxic model was established to simulate the pathological processes of PAH. qRT-PCR results showed that S1PR1 expression was significantly downregulated in endothelial cells after hypoxia treatment (*p* < 0.0001) (Figure 6A), suggesting that aberrant S1PR1 expression may be associated with endothelial dysfunction in PAH. To validate its function, S1PR1 was overexpressed in endothelial cells (*p* < 0.0001) (Figure 6B), and it was observed that the expression of the apoptosis-related protein Cleaved-Caspase 3 was significantly decreased in the overexpression group, while the expression of the anti-apoptotic protein Bcl-2 was significantly increased (Figure 6C). Concurrently, flow cytometry analysis confirmed that S1PR1 overexpression significantly reduced hypoxia-induced endothelial cell apoptosis (*p* < 0.0001) (Figure 6D). Furthermore, qRT-PCR results indicated that S1PR1 overexpression downregulated the expression of pro-inflammatory factors TNF-α, IL-1β, and IL-6 (*p* < 0.001) (Figure 6E), and significantly upregulated the expression levels of tight junction-associated proteins ZO-1 and Claudin-5 (Figure 6F).

Carbonic anhydrase, a transmembrane enzyme catalyzing reversible CO_2_ hydration and physiological pH regulation, also acts as a hypoxia-responsive gene and remains largely unexpressed in most normal tissues (Korkolopoulou et al., 2007; Elsawi et al., 2023). CAII upregulation in tumor endothelial cells both *in vitro* and *in vivo*, with CAII knockdown reducing tumor endothelial cell survival under lactic acidosis (Annan et al., 2019). Therefore, the present study further explored the influence of CA2 on endothelial cell function. Hypoxia significantly upregulated CA2 expression (*P* < 0.001, Supplementary Figure S1A), while CA2 knockdown produced effects opposite to those of S1PR1 overexpression (*P* < 0.001, Supplementary Figures S1B–F). These findings suggest that S1PR1 and CA2 may coordinately modulate endothelial homeostasis from opposing directions.

## 4 Discussion

PAH is a severe cardiopulmonary disorder characterized by a progressive increase in pulmonary vascular resistance and RHF. Its pathological process involves multiple mechanisms, including vasoconstriction, *in situ* thrombosis, and vascular wall remodeling (Ghofrani et al., 2025; Naeije et al., 2022). In recent years, the dysregulation of ion homeostasis has been recognized as a key factor driving the dysfunction of pulmonary artery endothelial cells and SMC. However, its systemic regulatory network and core effector molecules have not been fully elucidated (Chen et al., 2023; Perez-Vizcaino et al., 2021; Zhao et al., 2022). Focusing on IHRGs, we systematically revealed the diagnostic value of this gene set in PAH and its potential pathophysiological mechanisms by integrating multi-omics analysis, machine learning, and *in vitro* functional validation.

First, using WGCNA, we identified gene co-expression modules highly correlated with the PAH phenotype from the GSE117261 dataset. By intersecting these modules with the IHRGs gene set, we obtained 55 core candidate genes. Functional enrichment analysis revealed that these candidate genes are primarily involved in biological processes such as the regulation of calcium ion homeostasis, chemokine signaling pathways, and transmembrane transport. This indicated a close functional link and synergistic interaction between ion homeostasis dysregulation and immune-inflammatory responses, which together constitute a key molecular basis for the initiation and progression of PAH (Justus et al., 2024; Sundararaman and van der Vorst, 2021). Subsequently, three machine learning algorithms were employed for feature selection, ultimately identifying 9 core diagnostic genes, including ABCG2, CA2, and S1PR1. Among these, ABCG2, CA2, and S1PR1 demonstrated good diagnostic performance in two independent datasets, GSE117261 and GSE113439, validating their reliability and generalizability as potential biomarkers for PAH. Existing evidence highlights S1PR1, a key receptor in the S1P signaling axis, as crucial for maintaining pulmonary vascular endothelial barrier integrity and regulating vascular permeability; its dysfunction closely correlates with endothelial injury and vascular remodeling in PAH (31, 32). ABCG2, a transmembrane transporter protein involved in the excretion of various drugs and xenobiotics, whose downregulation may impair the endothelial cells’ ability to clear toxic metabolites, thereby exacerbating cellular stress and injury (Kukal et al., 2021; Homolya, 2021). CA2 plays a critical role in regulating intracellular pH and ion balance by catalyzing the hydration of carbon dioxide; its aberrant expression could disrupt cellular homeostasis and promote vascular remodeling (Rai et al., 2023; Raum et al., 2023). Collectively, ion homeostasis imbalance and endothelial dysfunction may constitute a critical pathological foundation for PAH.

In recent years, approaches based on systems biology and network analysis have offered novel insights into the complex molecular regulatory mechanisms underlying PAH. Recent studies leveraging knowledge graphs and biological network analyses have uncovered intricate crosstalk among metabolic disorders, G protein-coupled receptor signaling, the SRC/ERK1/2/AKT cascade, and the Activin/BMP signaling network in PAH. These signaling axes collectively modulate vascular remodeling, vasoconstriction, and endothelial barrier function (Weinstein et al., 2024). In the present study, transcriptomic data combined with machine learning algorithms were utilized to further screen core effector genes associated with ionic homeostasis from systemic networks, including S1PR1, CA2, and ABCG2. Despite adopting distinct analytical strategies, both studies indicate that the initiation and progression of PAH are not driven by the dysregulation of a single molecule, but rather by a multi-layered network imbalance encompassing metabolic regulation, ionic homeostasis, GPCR signaling, and vascular dysfunction. As a member of the GPCR superfamily, S1PR1 may serve as a molecular link connecting S1P signaling to endothelial barrier regulation. CA2 and ABCG2 are speculated to participate in PAH pathogenesis by modulating intracellular homeostasis and transmembrane substance transport. Collectively, the findings of this study supplement existing systems biology network models at the genetic level and further define promising candidate diagnostic biomarkers and directions for subsequent functional validation.

To gain deeper insights into the biological functions of these genes, we performed immune correlation analyses and regulatory network predictions. The analysis of immune characteristics revealed significant associations between these three genes and various aspects of the PAH immune microenvironment. Specifically, the expression level of S1PR1 was positively correlated with pDCs but negatively correlated with immune checkpoint molecules and CD8^+^ T cell infiltration, suggesting its potential involvement in PAH progression by regulating specific immune cell subsets and suppressing excessive immune activation (Chakraborty et al., 2019). The expression of ABCG2 was negatively correlated with the CCR signaling pathway, inflammation-promoting responses, and the infiltration levels of various immune cells (such as macrophages and B cells), indicating that this gene may function in anti-inflammation and the regulation of immune cell recruitment (El-Ashmawy et al., 2025). The expression of CA2 showed a negative correlation with para-inflammatory responses, further supporting its role in inhibiting non-specific inflammation. From an immunological perspective, these findings broaden the understanding of PAH pathogenesis, suggesting that PAH is a vascular disease and a “vascular inflammatory disease” closely associated with immune system dysfunction. To explore the upstream regulatory mechanisms governing the expression of diagnostic genes, this study predicted their TFs and miRNA regulatory networks. The analysis indicated that S1PR1 might be regulated by immune-related TFs such as IRF4 and potentially interacted with multiple miRNAs, suggesting its expression was co-regulated at both the transcriptional and post-transcriptional levels. To further elucidate the biological functions of the candidate genes, we constructed a PPI network based on the GeneMANIA database and performed functional enrichment analysis on the highly interconnected gene modules within the network. The results once again demonstrated that the related genes were significantly enriched in pathways involved in maintaining cellular material exchange and internal environment homeostasis, corroborating the findings from the previous WGCNA analysis.

To further explore the roles of S1PR1 and CA2 in PAH, *in vitro* experiments were conducted. Under hypoxic conditions, S1PR1 was significantly downregulated, and its overexpression markedly suppressed endothelial cell apoptosis, reduced inflammatory responses, and enhanced barrier-related protein expression, thereby functionally confirming its endothelial-protective effects. Concurrently, this study is the first to report significant upregulation of CA2 under hypoxia. CA2 knockdown reversed the phenotypic changes of increased apoptosis, enhanced inflammation, and compromised barrier function, exhibiting a regulatory trend opposite to that of S1PR1. This observation implicates CA2 in key regulatory processes governing endothelial homeostasis imbalance and provides experimental evidence for its potential mechanistic role in PAH.

In summary, this study, through bioinformatics and molecular biology approaches, screened core IHRGs with potential diagnostic value and elucidated the significant roles of these genes in the pathogenesis of PAH from multiple dimensions, including immune regulation, molecular networks, and cellular functions. Among them, IHRGs represented by S1PR1, ABCG2, and CA2 served as potential diagnostic biomarkers for PAH and ultimately influenced endothelial barrier function, survival, and inflammatory status by regulating ion homeostasis and immune-inflammatory responses. This study also has some limitations: First, although validated in multiple datasets, the identified diagnostic biomarkers still require further validation in prospective, large-scale clinical cohorts. Secondly, a cross-screening strategy incorporating multiple algorithms (SVM-RFE, Boruta and LASSO) was applied in this study to enhance the stability and reliability of the diagnostic model. Nevertheless, machine learning approaches tend to prioritize features with strong predictive performance, potentially missing several key nodes that exert vital biological functions yet exhibit subtype dependence or mediate network regulatory effects. For instance, although CAV1 was not included in the final core gene panel, it participates in the regulation of GPCR signaling, calcium homeostasis maintenance and endothelial function modulation, and may serve a critical role in the pathological network of PAH, including S1PR1-related cascades (Qin et al., 2026). Additionally, TBXAS1 is closely linked to PAH subtypes accompanied by thrombosis and oxidative stress-triggered vasoconstriction (Schildknecht et al., 2008). Accordingly, future investigations integrating systems biological network analysis, multi-omics integration and experimental validation are warranted to further dissect the roles of these excluded genes within the heterogeneous molecular networks of PAH. Moreover, functional mechanistic studies currently concentrate on the roles of S1PR1 and CA2 in endothelial cells; the specific functional mechanisms of ABCG2 in PAH require more in-depth exploration in future research. Finally, the regulatory relationships of TFs and miRNAs on the related genes predicted in this study need direct experimental validation using methods such as chromatin immunoprecipitation assays and dual-luciferase reporter assays.

## Data Availability

The original contributions presented in the study are publicly available. This data can be found in the Gene Expression Omnibus (GEO) repository under accession numbers GSE117261 and GSE113439.

## Supplementary material

SUPPLEMENTARY FIGURE S1 Effects of CA2 on endothelial cell biological functions under hypoxic conditions. **(A)** qRT-PCR analysis of CA2 expression in HPAECs under hypoxia. **(B)** qRT-PCR validation of CA2 knockdown efficiency. **(C)** WB analysis of Cleaved-Caspase 3 and Bcl-2 expression levels following CA2 knockdown under hypoxia. **(D)** Flow cytometry analysis of apoptosis following CA2 knockdown under hypoxia. **(E)** qRT-PCR analysis of TNF-α, IL-1β, and IL-6 expression levels following CA2 knockdown under hypoxia. **(F)** WB analysis of ZO-1 and Claudin-5 expression levels following CA2 knockdown under hypoxia. *, *p* < 0.05; **, *p* < 0.01; ***, *p* < 0.001; ****, *p* < 0.0001.

SUPPLEMENTARY TABLE S1 523 IHRGs.

SUPPLEMENTARY TABLE S2 The 55 genes obtained from the intersection of WGCNA and IHRGs analyses.

SUPPLEMENTARY TABLE S3 The 12 candidate genes selected by the SVM-RFE algorithm.

SUPPLEMENTARY TABLE S4 The 27 candidate genes selected by the Boruta algorithm.

SUPPLEMENTARY TABLE S5 The 16 candidate genes selected by LASSO regression.

SUPPLEMENTARY TABLE S6 The 9 genes intersecting the SVM-RFE, Boruta and LASSO regression algorithms.
